# Gene Evolutionary Trajectories and GC Patterns Driven by Recombination in *Zea mays*

**DOI:** 10.3389/fpls.2016.01433

**Published:** 2016-09-22

**Authors:** Anitha Sundararajan, Stefanie Dukowic-Schulze, Madeline Kwicklis, Kayla Engstrom, Nathan Garcia, Oliver J. Oviedo, Thiruvarangan Ramaraj, Michael D. Gonzales, Yan He, Minghui Wang, Qi Sun, Jaroslaw Pillardy, Shahryar F. Kianian, Wojciech P. Pawlowski, Changbin Chen, Joann Mudge

**Affiliations:** ^1^National Center for Genome Resources, Santa FeNM, USA; ^2^Department of Horticultural Science, University of Minnesota, St. PaulMN, USA; ^3^Section of Plant Biology, School of Integrative Plant Science, Cornell University, IthacaNY, USA; ^4^Biotechnology Resource Center Bioinformatics Facility, Cornell University, IthacaNY, USA; ^5^Cereal Disease Laboratory, United States Department of Agriculture – Agricultural Research Service, St. PaulMN, USA

**Keywords:** recombination, GC, meiosis, meiocytes, maize, codon usage, wobble, gene expression

## Abstract

Recombination occurring during meiosis is critical for creating genetic variation and plays an essential role in plant evolution. In addition to creating novel gene combinations, recombination can affect genome structure through altering GC patterns. In maize (*Zea mays*) and other grasses, another intriguing GC pattern exists. Maize genes show a bimodal GC content distribution that has been attributed to nucleotide bias in the third, or wobble, position of the codon. Recombination may be an underlying driving force given that recombination sites are often associated with high GC content. Here we explore the relationship between recombination and genomic GC patterns by comparing GC gene content at each of the three codon positions (GC_1_, GC_2_, and GC_3_, collectively termed GC_x_) to instances of a variable GC-rich motif that underlies double strand break (DSB) hotspots and to meiocyte-specific gene expression. Surprisingly, GC_x_ bimodality in maize cannot be fully explained by the codon wobble hypothesis. High GC_x_ genes show a strong overlap with the DSB hotspot motif, possibly providing a mechanism for the high evolutionary rates seen in these genes. On the other hand, genes that are turned on in meiosis (early prophase I) are biased against both high GC_x_ genes and genes with the DSB hotspot motif, possibly allowing important meiotic genes to avoid DSBs. Our data suggests a strong link between the GC-rich motif underlying DSB hotspots and high GC_x_ genes.

## Introduction

In eukaryotes, meiotic exchange of genetic information, or recombination, between homologous chromosomes is a critical step in generating genetic diversity required for adaptation. Recombination is also a crucial tool in plant improvement efforts. Local genome architecture is sculpted by the recombination process, and genome architecture, in turn, drives recombination. This interplay helps to create variability in genomic space, defining relatively stable and plastic genomic regions. This fluctuation in genomic stability is critical for balancing adaptation and stability on the phenotypic level.

Recombination has direct implications for GC patterns and *vice versa*. GC content refers to the percentage of guanine and cytosine bases in a DNA sequence, as opposed to adenine and thymidine bases. There have been many studies substantiating the positive correlation between recombination and GC content (Ikemura and Wada, 1991; Eyre-Walker, 1993; Fullerton et al., 2001; Galtier et al., 2001; Marais et al., 2001; Duret and Arndt, 2008; Haudry et al., 2008; Escobar et al., 2010; Muyle et al., 2011). Crossovers have been found to be correlated with high GC content in rat, mouse, human, zebrafish, bee, and maize at a broad scale (Jensen-Seaman et al., 2004; Beye et al., 2006; Gore et al., 2009; Backstrom et al., 2010; Giraut et al., 2011), while other studies detected strong correlation only at a fine scale (∼5 kb for yeast, ∼15–128 kb for human) and rather weak correlation at a broad scale (∼30 kb for yeast, ∼1 Mb for human; Gerton et al., 2000; Myers et al., 2006; Marsolier-Kergoat and Yeramian, 2009).

In plants, correlation of recombination and GC content has been demonstrated in multiple species (Haudry et al., 2008; Escobar et al., 2010; Muyle et al., 2011). A study examining three different grasses (rice, maize, and *Brachypodium*) revealed significant correlation of the local recombination rate with high GC content, especially in the wobble codon position (third position in the codon; Serres-Giardi et al., 2012). This was in contrast to prior studies performed with lower levels of resolution, which found only weak correlation in maize and none in rice or *Brachypodium* (Gore et al., 2009; Tian et al., 2009; Huo et al., 2011). However, negative correlation between crossovers and high GC content was reported for *Arabidopsis* (Drouaud et al., 2006) in spite of the fact that a crossover motif has been identified that has high GC content every third nucleotide (Wijnker et al., 2013).

**FIGURE 1:**
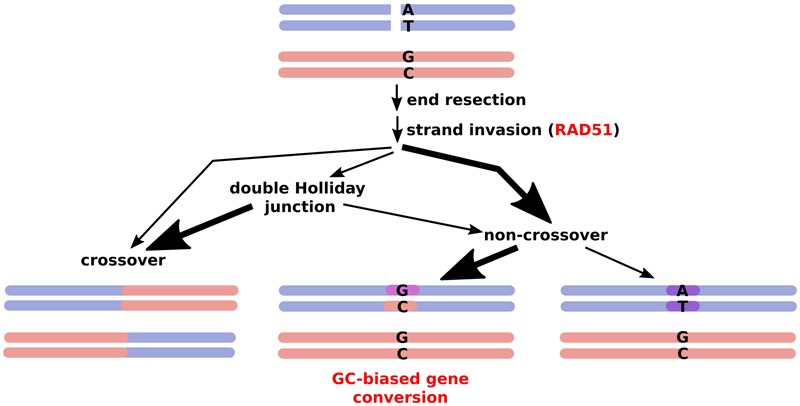
**Outcomes of meiotic recombination.** A double strand break is processed via different pathways, with very few DSBs resulting in actual crossovers while a majority are resolved via gene conversion, frequently inserting a GC bias due to mismatch repair. Thick arrows represent the major routes.

While most studies agreed on a positive correlation between recombination and high GC content, the cause for this has been disputed (Duret and Arndt, 2008; Marsolier-Kergoat and Yeramian, 2009; Giraut et al., 2011). Possible reasons suggested for the high GC/crossover correlation include selection on codon usage, mutational bias, or GC-biased gene conversion (Eyre-Walker and Hurst, 2001; Duret and Galtier, 2009). The latter is seen as the most likely cause for GC enrichment (**Figure 1**), and has been suggested for diverse organisms such as yeast, mammals, and birds (Webster et al., 2006; Duret and Arndt, 2008; Mancera et al., 2008; Nabholz et al., 2011). A study by Birdsell (2002) presented compelling evidence demonstrating a highly significant positive correlation between GC in the wobble position and recombination within 6,143 ORFs analyzed in the yeast (*Saccharomyces cerevisiae*) genome. This study also showed a significant correlation between recombination and the mean GC content of the first and second codon and recombination, but not to the same extent as with the GC content in the third, or wobble, position (Birdsell, 2002).

In biased gene conversion, repair tracts at recombination sites that are not necessarily resolved into crossovers favor changes to GC over AT (Eyre-Walker, 1993; Birdsell, 2002). This can occur simply by using the mismatch repair machinery (Marais, 2003) or by repair of double strand breaks (DSBs) in GC-poor alleles with GC rich ones, which occurs in human but not in yeast (Duret and Arndt, 2008; Marsolier-Kergoat and Yeramian, 2009; Marsolier-Kergoat, 2011). In yeast, GC-biased gene conversion was found specifically at crossovers, not at DSBs that did not involve gene conversion and did not result in a crossover, and was shown to be due to mismatch repair rather than base excision repair or DSB repair (Lesecque et al., 2013). Even within-species variation in GC content is partly related to recombination, being correlated in mammals, birds, and yeast (Birdsell, 2002; Duret and Arndt, 2008; Nabholz et al., 2011). Notably, it has been proposed for angiosperms, that recombination and GC-biased gene conversion drives gene GC content patterns, even forming a 5′–3′ gradient along many genes (Glémin et al., 2014). However, GC-biased gene conversion might be attenuated in inbreeding species such as selfing grasses leading to no apparent correlation (Giraut et al., 2011).

Triplets of the four major nucleotide bases (guanine, cytosine, adenine, and thymine/uracil) can be combined in 64 (4^3^) different ways but encode for only 20 amino acids and the stop codon. Thus, there is redundancy in the genetic code, resulting in triplets (codons) that differ but are synonymous concerning their matching amino acid. GC content, mutations, and selection pressure are critical for the evolution of heterogeneity in codon usage (Sharp et al., 1988; Sharp and Matassi, 1994; Karlin and Mrazek, 1996; Sueoka and Kawanishi, 2000). By now, it has been well documented that synonymous codon usage varies significantly among genomes and, in addition, among different genes within any given genome (Grantham et al., 1980; Sharp et al., 1988; Wang and Hickey, 2007). One implication of higher GC content from silent codon positions can be a general increased expression due to higher stability of mRNA, as demonstrated in mammals (Kudla et al., 2006). Intra- and inter-genomic deviations in codon usage can be attributed to many factors. In the case of prokaryotes and unicellular organisms, it is thought to be a result of natural selection during which protein production is optimized (Gouy and Gautier, 1982; Sharp and Li, 1986; Shields et al., 1988; Sharp et al., 2005). Likewise, in some higher eukaryotes, there is also evidence that codon bias may occur due to selection for translational efficiency (Shields et al., 1988; Stenico et al., 1994). Particularly, there is a positive correlation between the complementarity of the codons in highly expressed genes and the anticodons of the most abundant tRNAs (Ikemura, 1981; Moriyama and Powell, 1997; Kanaya et al., 1999).

Numerous studies have been conducted on codon usage in plant species including the model plants Arabidopsis *(Arabidopsis thaliana*), rice (*Oryza sativa*), and a moss (*Physcomitrella patens*), as well as in poplar, citrus and true grass (Poaceae/Gramineae) species (Chiapello et al., 1998; Liu et al., 2003; Guo et al., 2007; Ingvarsson, 2008; Xu et al., 2013). In addition to the reported factors involved in codon usage bias, higher average GC content in monocot compared to dicot genomes plays an important role in dictating codon usage differences between the two groups of plants (Fennoy and Bailey-Serres, 1993; Carels and Bernardi, 2000). These compositional variations are illustrated by an abundance of genes with high GC levels in the third codon position in monocots (Matassi et al., 1989). The third position in the codon, also referred to as the wobble position, is less biased for the amino acid than the other two bases and accounts for most of the degeneracy of the genetic code. The wobble position functions as a marker for GC richness, and the frequency of GC nucleotides at the third position is defined as GC_3_ (Chiapello et al., 1998; Tatarinova et al., 2010; Elhaik et al., 2014).

In some organisms, two classes of genes can be distinguished by their high or low GC_3_ content (Carels and Bernardi, 2000; Tatarinova et al., 2010). This trait is thought to be ancestral to monocots, with some lineages losing the bimodal GC_3_ distribution (Clément et al., 2014). These two GC_3_ classes in monocots experience divergent evolutionary pressure and contain different functional categories of genes. High GC_3_ genes within monocots were mainly categorized into functions involving electron transport or energy pathways, response to biotic and abiotic stressors and signal transduction (Carels and Bernardi, 2000; Tatarinova et al., 2010). High GC_3_ genes can be turned on quickly and experience accelerated evolution (Tatarinova et al., 2010).

Kawabe and Miyashita (2003) went beyond GC_3_ content and described GC content in the first, second, and third codon positions for seven plant species, including monocots and dicots, showing that GC_1_ and GC_2_ in addition to GC_3_ were significantly higher in monocots than dicots. The differences in GC content were the largest in the third codon position, followed by the first and then the second position. Studies by Cruveiller et al. (2000, 2004) report a linear correlation between genic GC_2_ and GC_3_ levels, which is found in species as distant as human and bacteria.

As part of a larger project, we have explored the genomic landscape of meiosis, specifically looking at early prophase I in isolated plant meiocytes, the cells which undergo meiosis and recombination. Previous publications have focused on the functional aspects of gene expression (Chen et al., 2010; Dukowic-Schulze et al., 2014a,b) and the landscape of DSB sites and its distribution (He et al., in review). This article focuses primarily on the implications of recombination on genome evolution, as measured by GC patterns, and *vice versa*.

Recombination occurs during meiosis, specifically during prophase I when DSBs are formed and homologous chromosomes pair and recombine (Padmore et al., 1991; Sheehan and Pawlowski, 2012; Dukowic-Schulze et al., 2014b). While DSBs initiate recombination and are a necessary prerequisite, most DSBs will not be resolved into crossovers. Two *Arabidopsis* motifs associated with crossovers have been identified, including one that showed high GC content every three nucleotides (Wijnker et al., 2013). In maize, a previously identified variable motif underlying genic DSB hotspots is GC-rich and also shows high GC periodicity every three nucleotides, reminiscent of GC periodicity within the codon (He et al., in review).

In this study, we used maize as a model to better under stand the relationship between genome architecture and recombination. We examine the interplay between genome evolution (including divergent evolutionary trajectories within a single genome), GC patterns, and recombination initiation in maize. Specifically, we address whether the GC-rich, three nucleotide-periodic motif underlying DSB hotspots in maize correlates with GC_3_ or other codon-driven GC patterns. In addition, we address how meiotic genes fit into the DSB and GC landscapes. Concurrently, we extend present knowledge of GC_1_, GC_2_, and GC_3_, collectively termed GC_x_, in *Zea mays*, by examining the relationships among them. In short, we aim to learn whether DSB-associated motifs with high GC content, particularly at every third nucleotide, could be the driving force behind bimodal GC patterns that split the maize genome into labile and stable evolutionary trajectories.

## Materials and Methods

### Reference Genome

All analyses used the B73 maize reference genome version RefGen_v2 to match previous expression analysis. The filtered gene set (annotation set 5b, gff format) was used for gene annotation.

### Differential Expression Analysis

Samples, sequence, and differential expression analyses are described in Dukowic-Schulze et al. (2014a,b,c). For this analysis, a less stringent dataset was used with a significance cutoff for calling differential expression increased from *p* = 0.01 to *p* = 0.05.

### Double Strand Break Hotspots

Using ChIP-seq with antibodies against the RAD51 protein as described in He et al. (2013), the DSB hotspot motif was identified with the sequence GVSGRSGNSGRSGVSGRSG (He et al., in review). The motif was identified from ∼900 genic hotspot regions that did not contain transposable elements. Copies of the motif were identified using the rGADEM package (Li, 2009) to re-scan these genic hotspot regions for matches to the position weight matrix of the motif using a stringency of 80%.

### GC Calculations

GC, GC_1_, GC_2_, and GC_3_ were calculated using custom Perl scripts. For GC_1_, GC_2_, and GC_3_, calculations for each gene were performed on the sequence that contributes to the protein (coding domain sequences (CDSs), and redundancies removed where CDSs overlapped. The phase of each CDS, defined as the number of nucleotides that need be removed from the beginning of the CDS to find the first base of the next codon, was taken into account. GC_1_ represents the GC content of the first nucleotides, GC_2_ the content of the second nucleotides, and GC_3_ the content of the third nucleotides of all codons in a gene. Genic GC was calculated for exons only (CDSs) as well as for exons together with introns in the pre-mRNA.

### Pathway Enrichment Analysis

agriGO was used to perform gene ontology (GO) enrichment studies (Du et al., 2010) using singular enrichment analysis to identify enrichment compared to the *Z. mays* reference. Advanced statistical options include Fisher’s exact test and, in order to perform multi-comparison adjustment with the large input dataset, the Benjamini–Hochberg correction method (Benjamini and Hochberg, 1995). A significance value of 0.05 was used to obtain lists of enriched GO terms unless the input gene list was large, in which case we focused on the most significant terms (*p* = 0.01). This did not alter the nature of the functionalities that were enriched for within the analyses. In order to consolidate the large list of GO terms, REVIGO was used (Supek et al., 2011). REVIGO uses a simple hierarchical clustering procedure to remove redundant terms, summarize related terms, and visualize the final set of GO terms.

### Plotting and Statistical Analyses

Plotting was done in R Statistical Package 3.2.0 and two-sided chi-square tests performed in Microsoft Excel v 14.6.4.

## Results

### GC Patterns in Maize Genes Show Bimodal Peaks with a Strong Bias in the Third Codon Position

**FIGURE 2:**
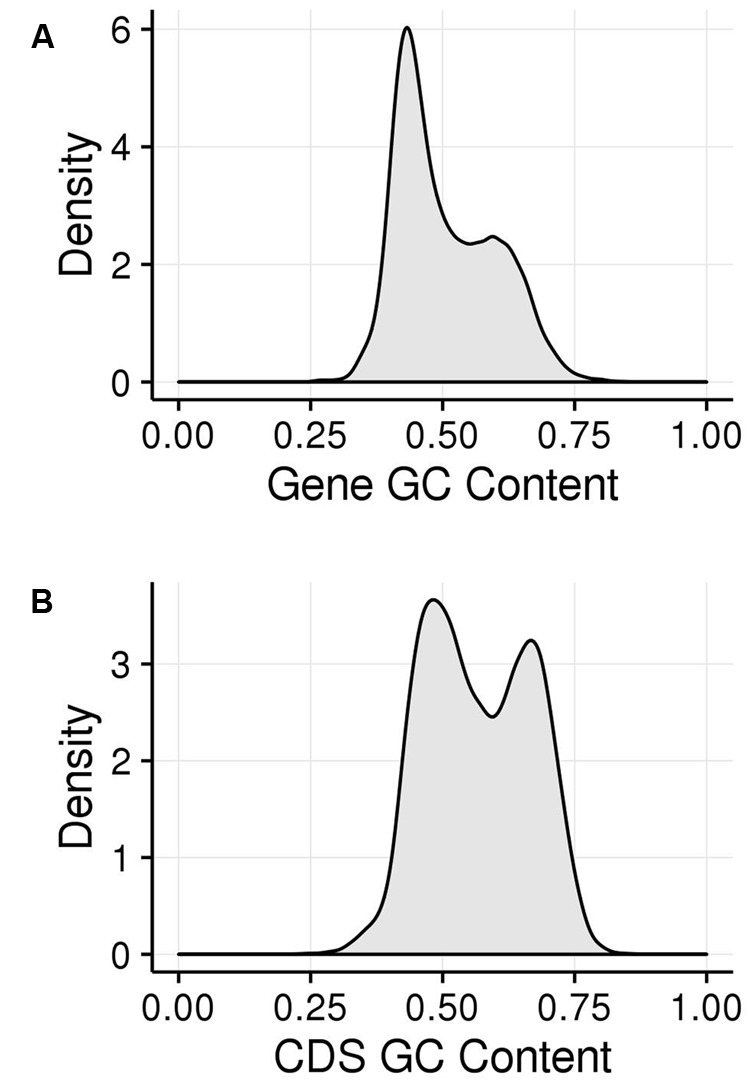
**GC patterns within maize coding regions shown in kernel density plots. (A)** Histogram of GC content of genes (including exons and introns). **(B)** Histogram of GC content of genes (just CDS sequence). The area under the curve represents the probability of getting a value in a given range of GC content, with the area under the entire curve equal to 1.

We examined the GC content of maize genes and their CDSs (**Figure 2**). The GC content of maize genes shows a bimodal peak, indicating that there are two classes of genes in the maize genome that are differentiated by GC content. This matches previous observations (Duret et al., 1995; Carels and Bernardi, 2000; Lescot et al., 2008; Paterson et al., 2009) and hold true both when calculated across genes, including introns (**Figure 2A**), and when calculated just across CDSs (**Figure 2B**). Including introns and untranslated regions (UTRs; **Figure 2A**) appears to bias genes toward the lower GC content class compared to the pattern using only CDS, though both classes are clearly visible. This suggests that the high GC content is maintained preferentially in the CDS regions. As discussed above, a strong bias in GC content at the third codon position has been shown to contribute to the bimodality of maize genic GC content but bimodality has been shown for all three codon positions. We took a comprehensive approach and examined GC content in the first (GC_1_), second (GC_2_), and third (GC_3_) codon positions, collectively termed GC_x_.

**FIGURE 3:**
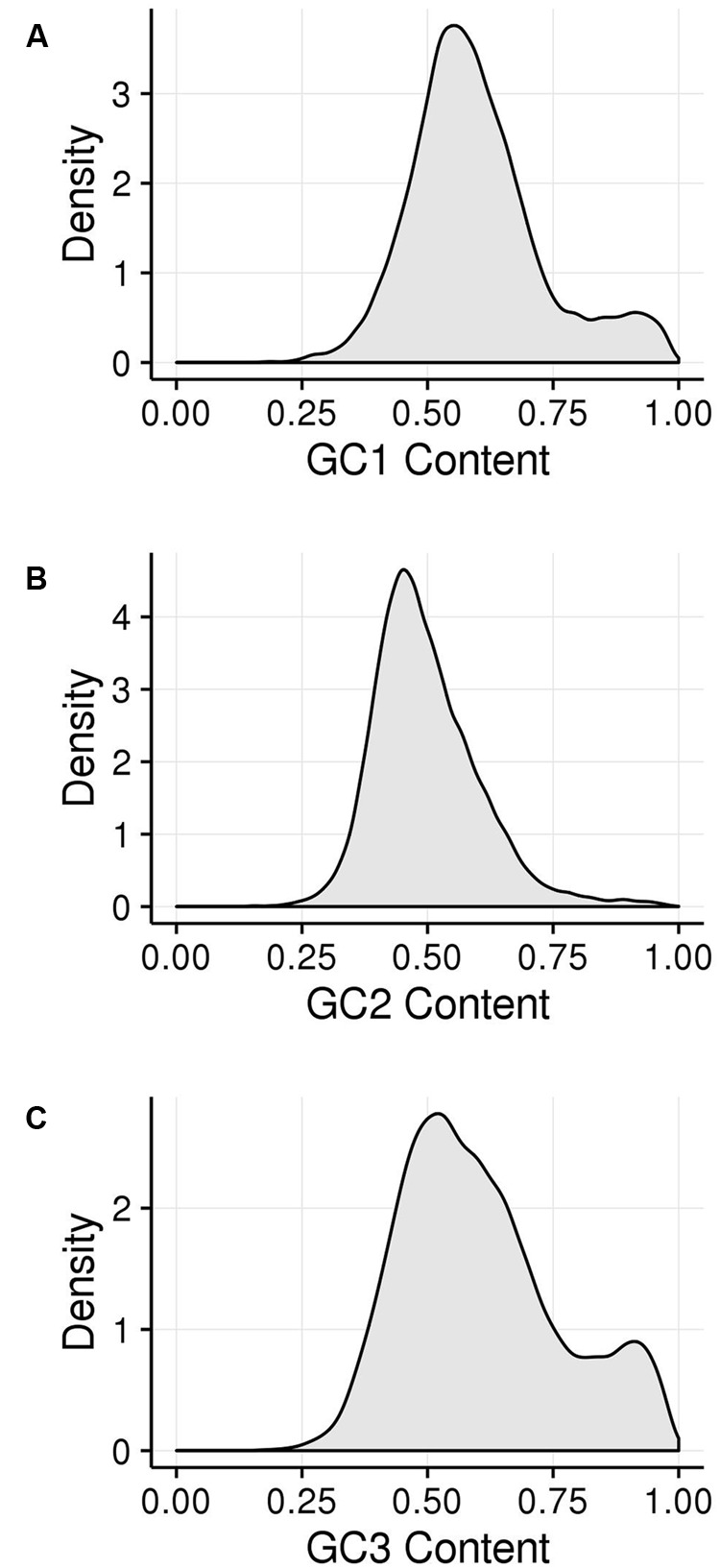
**Kernel density plots of GC_x_ distribution patterns of gene CDSs. (A)** GC_1_. **(B)** GC_2_. **(C)** GC_3_.

**FIGURE 4:**
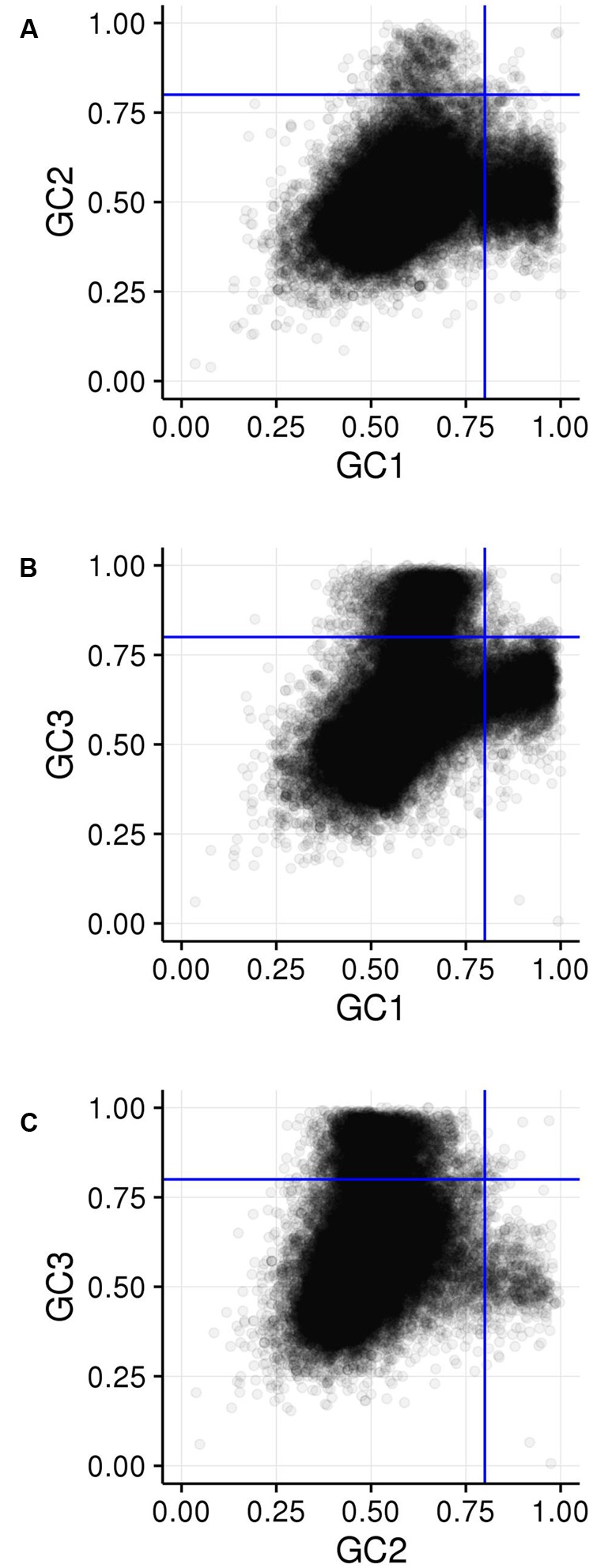
**Comparison of GC_x_ distributions. (A)** GC_1_ vs. GC_2_. **(B)** GC_1_ vs. GC_3_. **(C)** GC_2_ vs. GC_3_. Blue lines show the 80% cutoff separating the high and low GC_x_ classes.

GC_1_, GC_2_, and GC_3_ were calculated for all genes and the distribution plotted (**Figure 3**). A bimodal distribution was easily apparent for GC_1_ and GC_3_ (GC content peaks around 50–55% and 90–95%) where both GC content classes had higher GC content than the overall genic GC content (see **Figures 2** and **3**), regarding both the intron-inclusive (40–45% and around 60%) and the intron-exclusive CDS analysis (∼50% and 70–75%; **Figure 3**). In contrast, GC_2_ analysis showed had only one discernible peak of far lower GC content (around 45%), with a long tail to the right, with possibly a very shallow second peak showing high GC content in the same range as that seen in GC_1_ and GC_3_. As expected, the peak containing high GC content genes was most pronounced in the third position. A cutoff of ≥80% (Tatarinova et al., 2010) was used for all GC_x_ conditions to identify the class of high GC_x_ content genes among the 39,656 genes present in *Z. mays* (**Table 1**). Among the high GC_x_ content classes, the high GC_3_ content class had the most genes at 5,719, followed by the high GC_1_ content class at 3,647 genes. GC_2_ had the fewest with 629 genes (**Table 1**). There was almost no overlap between each of the high GC_x_ classes (**Figure 4**).

**Table 1:** Counts of high and low GC_x_ genes.

GC_x_	High GC_x_ genes (≥80%)	Low GC_x_ genes (<80%)
GC_1_	3,647	36,009
GC_2_	629	39,027
GC_3_	5,719	33,937

### GC Patterns in Genic Double Strand Break Hotspot Motifs Show High GC Every Third Nucleotide

To gain insight on patterns possibly shaping bimodality and GC_x_ classes, we looked at copies of the DSB hotspot motif (hereafter referred to as “motifs”) in genic regions (He et al., in review). Of 3,423 DSB genic hotspot motifs, 2,143 fell into 544 genes. Of these, 1,909 motifs fell into 811 CDSs of 511 genes, and the remaining 234 motifs occurred in introns and 5′ and 3′ UTR regions. Maize genes are, on average, less than 50% coding sequence (1,153.7 nt of intron sequence for every 1 kb of CDS), yet 89% of the motifs occurred in the CDS. This indicates a strong preference for copies of the DSB hotspot motifs to occur in the protein-coding portion of the gene (chi-square *p*-value = 0). Of the 544 genes containing hotspot motifs, 84% contained more than one motif instance indicating the propensity for them to cluster (**Table 2**).

**Table 2:** Frequencies of double strand break hotspot motifs per gene.

Motifs per Gene	Number of Genes
1	85
2	107
3	92
4	75
5	58
6	45
7	32
8	20
9	13
10	5
11	6
12	5
15	1
16	1

**FIGURE 5:**
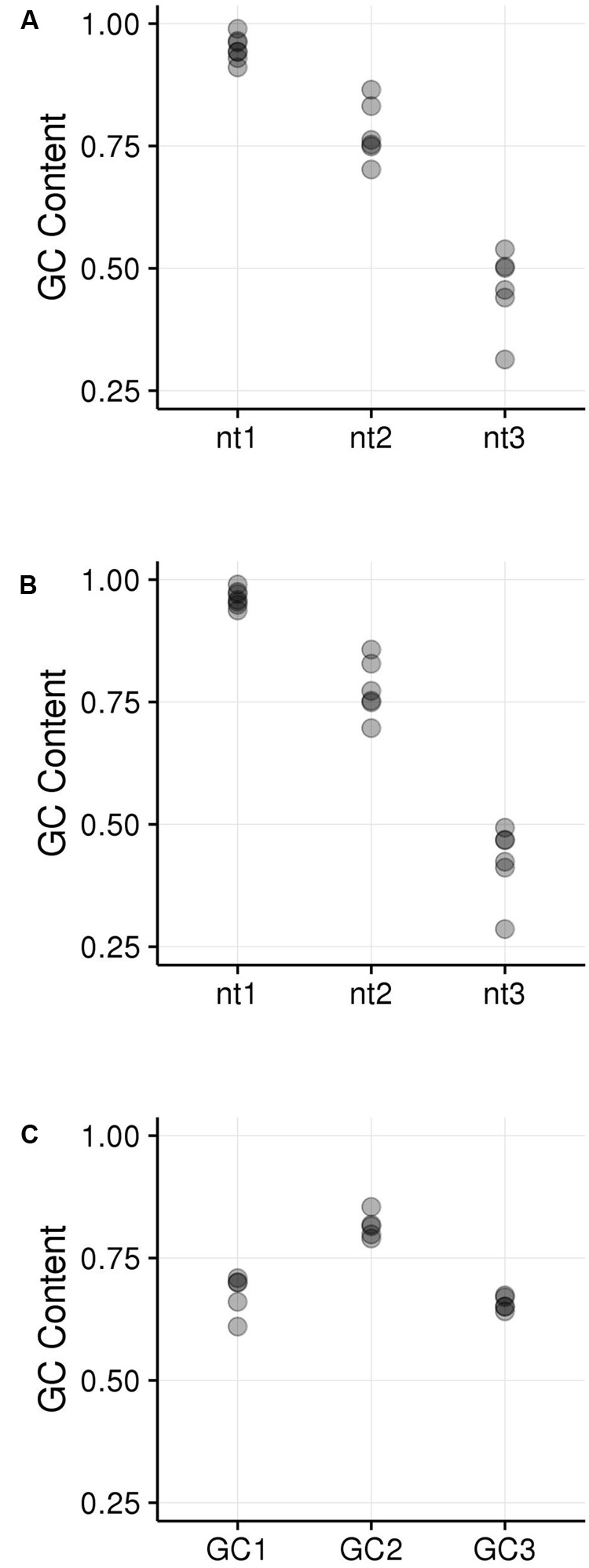
**GC content within hotspot motifs was compared between 3 nt-based periodicity groups across all motif instances. (A)** All hotspot motifs are plotted in groups of every third nucleotide starting with nt1, nt2, or nt3. The nt1 group includes GC content calculated across all motifs for nucleotide positions 1, 4, 7, etc. Each position was analyzed separately. Likewise, the nt2 and nt3 groups have GC content calculated for every third nucleotide position starting with nt2 and nt3, respectively. **(B)** All genic hotspot motifs are plotted in groups of every third nucleotide as in **(A)**; **(C)** The reading frame of the motif was determined in order to place nucleotides into the GC_1_, GC_2_, and GC_3_ categories. Then, GC content was calculated for the GC_1_, GC_2_, and GC_3_ position within the motif, trimming the end nucleotide overhangs that resulted when shifting motifs to line up GC_1_, GC_2_, and GC_3_ positions.

GC content of the hotspot motif (GVSGRSGNSGRSGVSGRSG) demonstrates a 3 nt-based periodicity (He et al., in review). While none of the positions are perfectly conserved, the G’s at every third nucleotide have information content values that are much higher than at any other residue and range from approximately 1.2 to 1.7. Indeed, every third position starting with the first nucleotide is nearly always “G.” We determined GC content across positions 1–19 of all underlying motifs. GC content per nucleotide position, averaged across all motif occurrences, was calculated for all hotspot motifs as well as only those hotspot motifs falling into genes. Every third nucleotide was grouped into a separate periodicity group (i.e., group 1 = nucleotide positions 1, 4, 7, 10, 13, 16, 19; group 2 = nucleotide positions 2, 5, 8, 11, 14, 17; group 3 = nucleotide positions 3, 6, 9, 12, 15, 18). These groups are framed relative to the start position of the motif and not necessarily to the coding frame of the containing gene (**Figures 5A,B**). Pairwise differences between GC content of these groups all showed significant differences (**Table 3**).

**Table 3:** Comparison of GC content between 3 nt-based periodic nucleotide groups of DSB motifs within DSB hotspots and genic DSB hotspots.

	Comparisons	*F*-test for equal variance	Equal variance (homoscedastic), two-tailed *t*-tests	Unequal variance (heteroscedastic), two-tailed *t*-tests
All motifs (position within motif)	nt1 vs. nt2	0.06315758		0.000426348
	nt2 vs. nt3	0.548540329	1.41343E-05	
	nt1 vs. nt3	0.016068299		7.88762E-06
Genic motifs (position within motif)	nt1 vs. nt2	0.012207227		0.000329802
	nt2 vs. nt3	0.596027462	3.77664E-06	
	nt1 vs. nt3	0.003212561		5.4902E-06
Genic motifs (position within codon)	GC_1_ vs. GC_2_	0.348471769	0.000199458	
	GC_2_ vs. GC_3_	0.288858144	1.7E-06	
	GC_1_ vs. GC_3_	0.058398065		0.38307282

*An *F*-test was performed to test the hypothesis of equal variances followed by one of two *t*-tests based on the outcome of the *F*-test. See the legend for **Figure 5** for a detailed description of the groups. All comparisons were done either as a homoscedastic or a heteroscedastic *t*-test, depending on the outcome of the *F*-test.*

**FIGURE 6:**
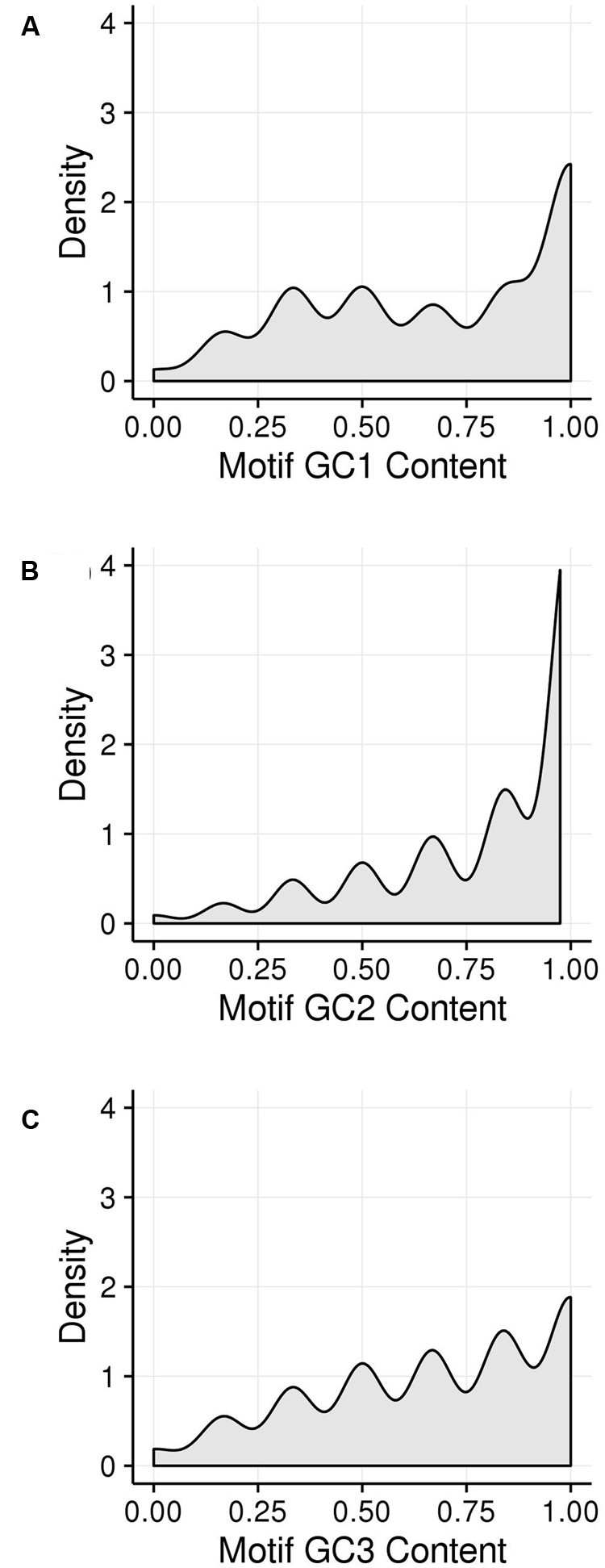
**Density plot of GC_x_ content of individual motifs. (A)** GC_1_, **(B)** GC_2_, **(C)** GC_3_.

Because the periodic groups of the DSB hotspot motifs are not necessarily in frame with a gene’s coding frame, we analyzed GC_x_ content of the motifs by identifying its coding frame and adjusting it from motif coordinates (relating to the beginning of the motif) to GC_x_ space (relating to the coding frame of the gene that contains the motif). Indeed, motifs fell into all three possible coding frames, with 868 matching the gene’s coding frame (i.e., the first base of the motif is also the first position of a codon), 389 starting on the second position of the codon, and 1,094 starting on the third position of the codon. Nucleotides within motifs were then placed into GC_x_ groups based on their position within the codon rather than in the motif. The GC_2_ group within motifs showed significantly higher GC content than either the GC_1_ or GC_3_ groups and was the only group that overlapped the high GC_x_ range (≥80%; **Figure 5C**). This is particularly interesting when considering that genes with high GC_2_ content are far less frequent than those with high GC_1_ or GC_3_ (**Figure 3**). Given these patterns, we tested whether high GC_x_ on a gene level is correlated with high GC_x_ of the contained motif. Genes containing DSB hotspot motifs were indeed significantly overrepresented for high GC_x_ genes (**Table 4**). Around 70% (380/544) of the genes containing DSB hotspot motifs fall into at least one peak of high GC_x_ content (i.e., GC_1_, GC_2_, and/or GC_3_). Of these, only three had high GC_x_ content in more than one category, and only one had high GC_x_ content in all three. The number of DSBs that fall into each of the high GC_x_ categories mirrors the size of each of the high GC_x_ content peaks. Twenty-four percent of genes containing DSB hotspots have high GC_1_ content, 5% have high GC_2_ content, and 42% have high GC_3_. When looking at the GC_x_ content of individual DSB hotspot motifs, as opposed to looking at them collectively as done above, a large fraction of the motifs are GC-rich (≥80%), including many with 100% GC content (**Figure 6**). Indeed, more than a quarter of motifs have 100% GC_1_ and GC_3_ content and more than half have 100% GC_2_ content. It is thus not surprising that genes containing the motifs also tend to be have high GC_x_ content.

**Table 4:** Chi-square test to determine whether GC_x_ high and low categorization is randomly distributed within the 544 DSB motif-containing genes.

	High GC_x_ observed (expected)	Low GC_x_ observed (expected)	*p*-value
GC1	130 (50)	414 (494)	1.79998E-32
GC2	28 (9)	516 (535)	2.97987E-11
GC3	227 (78)	317 (466)	1.87802E-73

### Genes with High and Low GC_x_ Content Show Functional Differentiation

Among the 39,656 genes annotated in the maize reference genome, 9,861 genes (25%) are in a high GC_x_ content peak. However, there is very little overlap between high GC_x_ genes in the GC_1_, GC_2_, and GC_3_ categories (**Figure 4**). Indeed, overlap rates are significantly less than expected given random sampling (**Table 5**). The lack of overlap is especially stark between the high GC content peaks of GC_1_ and GC_3_ (**Figure 4B**) where very few genes simultaneously have high GC_1_ and high GC_3_ content (≥80%), while many genes have a strong positive correlation when being of both low GC_1_ and GC_3_ content (<80%). Of 526 genes that would be expected to fall into the high GC_3_ content peak given random sampling of 3,647 high GC_1_ genes from the maize genome, only 96 (18% of expectation) fall into the high GC_3_ content peak. Furthermore, most of the genes that do show high GC_1_ and GC_3_ content are limited to GC_x_ contents close to the 80% cutoff in one or both GC_x_ categories, indicating they may be part of the upper tail of the lower GC_x_ content peak that merges into and is indistinguishable from the high GC_x_ content peak (**Figure 3**). Taken together, there seems to be a selection against high GC_x_ content in more than one codon position.

**Table 5:** Chi-square test to determine whether other GC_x_ classes are randomly distributed within each of the high GC_x_ classes.

	High GC_x_ observed (expected)	Low GC_x_ observed (expected)	*p*-value
Among high GC_1_ genes			
GC_2_	26 (58)	3,621 (3,589)	**2.43E-05**
GC_3_	96 (526)	3,551 (3,121)	**2.57E-91**
Among high GC_2_ genes			
GC_1_	26 (58)	603 (571)	**1.11E-05**
GC_3_	26 (91)	603 (538)	**2.06E-13**
Among high GC_3_ genes			
GC_1_	96 (526)	5,623 (5,193)	**3.59E-86**
GC_2_	26 (91)	5,693 (5,628)	**7.44E-12**

*Significant *p*-values allowing rejection of the null hypothesis (*p* ≤ 0.05) are in bold.*

When genes in the low GC_3_ class were subjected to agriGO analysis, housekeeping pathways were enriched, such as genes involved in cellular processes, metabolic processes, protein metabolism, protein modifications, biosynthetic pathways, localization, and transport. Genes responsible for transcription and transcriptional regulation processes were also categorized as low GC_3_. Genes in the high GC_3_ class fell into functions implicated in the immune system and stress responses including adaptive immunity and response to wounding. Genes involved in sexual reproduction were also enriched in the high GC_3_ class though this did not appear to translate specifically to enrichment of meiotic recombination genes. In short, our results indicate more generalized or housekeeping functions for low GC_3_ genes, SOS-type functions for high GC_3_ genes, and are very similar to those seen in other studies (Carels and Bernardi, 2000; Tatarinova et al., 2010).

In the case of GC content of the first nucleotide of each codon, 36,009 genes were categorized with low GC_1_ (<80%) and 3,648 genes with high (≥80%) GC_1_ content. GO analysis on GC_1_ gene classes showed similar trends to the GC_3_ classes. Low GC_1_ genes described functions such as cellular protein modification process, protein localization, macromolecule metabolism (primary and cellular metabolism), including lipid and phosphorus metabolism, gene expression, and cell death. High GC_1_ genes were implicated in functions such as response to stress, DNA packaging, regulation of biological quality, adaptive immune response, fatty acid metabolism, and response to stimulus.

When all genes in *Z. mays* were examined for the GC content in the second nucleotide of each codon, even though there were significantly fewer genes with high GC content compared to high GC_1_ and GC_3_ content, the general trends observed in GC_1_ and GC_3_ were seen in GC_2_ for the low (39,027 genes) and high (630 genes) GC_2_ classes with some subtle exceptions. To elaborate, low GC_2_ genes were implicated in ontologies such as protein metabolism, localization, RNA metabolism, lipid metabolism, and intracellular transport. The 630 high GC_2_ genes, however, were categorized into different ontologies compared to the high GC_1_ and high GC_3_ gene sets. High GC_2_ genes were involved in cell-wall organization, transmembrane transport, lipid, carbohydrate, and phosphorus metabolism, G-coupled signaling pathways, protein phosphorylation, post-translational modification, sexual reproduction, and localization type functions. The low number of genes in the high GC_2_ class may have lowered our power to detect some of the classes seen in high GC_1_ and GC_3_ genes.

Overall, high GC_x_ genes tend to play a role in stress and adaptive responses as well as sexual reproduction. The low GC_x_ genes tend to be biased toward more generalized functions.

### Genes Up-Regulated in Meiocytes Are Underrepresented for DSB Motifs

To study correlations between DSB motif presence, GC_x_ patterns and gene expression, we examined genes that contained DSB hotspot motifs regarding their expression level in meiocytes compared to anthers or seedlings (**Table 6**). Genes with DSB hotspot motifs were down-regulated in meiocytes at a rate expected given independence of down-regulated genes and motifs. However, meiocyte up-regulated expression of DSB hotspot motif-containing genes occurred at a lower frequency than expected randomly. This bias is significant in the meiocytes vs. seedlings comparison but not in the meiocytes vs. anthers comparison, likely due to the large overlap of gene expression patterns between meiocytes and anthers (Dukowic-Schulze et al., 2014b).

**Table 6:** Chi-square test to determine whether up- or down-regulated genes in meiocytes are randomly distributed within the 544 motif-containing genes.

Differential expression in meiocytes		Yes	No	*p*-value
Meiocyte-specific expression	Comparison tissue	Observed (expected)	Observed (expected)	
Up-regulated	Anther	2 (3)	542 (541)	0.712147988
Up-regulated	Seedling	18 (44)	526 (500)	**3.57589E-05**
Down-regulated	Anther	2 (3)	542 (541)	0.506157854
Down-regulated	Seedling	62 (57)	482 (487)	0.465276348

*Significant *p*-values allowing rejection of the null hypothesis (*p* ≤ 0.05) are in bold.*

We approached our analysis from another angle, and looked at GC_3_ distribution of all genes with any expression in meiocytes in B73. When considering all genes that are expressed in meiocytes, GC_3_ distribution does not differ from GC_3_ distribution across all genes (**Table 7**). However, when zooming in specifically on genes that are up- or down-regulated in meiocytes compared to seedlings or anthers (which contain meiocytes), GC-based trends become clear. Genes up-regulated in meiocytes tend to have low GC_3_ content (<80%) while genes down-regulated in meiocytes tend to have high GC_3_ (≥80%; **Table 7**). Similar trends are seen with GC_1_ and GC_2_ (**Table 7**).

**Table 7:** Chi-square test on B73 genes that are expressed in or up- or down-regulated in meiocytes compared to anthers or seedlings.

Meiocyte expression	GC_x_	Low GC_x_ observed (expected)	High GC_x_ observed (expected)	*p*-value
All expressed genes	GC_1_	22,023 (21,981)	2,184 (2,226)	0.3477
All expressed genes	GC_2_	23,852 (23,823)	355 (384)	0.1363
All expressed genes	GC_3_	20,648 (20,716)	3,559 (3,491)	0.2136
Up vs. anthers	GC_1_	180 (172)	9 (17)	**0.0349**
Up vs. seedlings	GC_1_	3,002 (2,938)	234 (298)	**1.0900E-04**
Down vs. anthers	GC_1_	192 (211)	40 (21)	**2.2300E-05**
Down vs. seedlings	GC_1_	3,560 (3,759)	580 (381)	**8.5100E-27**
Up vs. anthers	GC_2_	186 (186)	4 (3)	0.5596
Up vs. seedlings	GC_2_	3,200 (3,185)	36 (51)	**3.1000E-02**
Down vs. anthers	GC_2_	224 (228)	8 (4)	**2.3200E-02**
Down vs. seedlings	GC_2_	4,029 (4,074)	111 (66)	**1.7100E-08**
Up vs. anthers	GC_3_	179 (162)	10 (27)	**0.0004**
Up vs. seedlings	GC_3_	2,855 (2,769)	381 (467)	**1.7800E-05**
Down vs. anthers	GC_3_	167 (199)	65 (33)	**3.7800E-09**
Down vs. seedlings	GC_3_	3,117 (3,543)	1,023 (597)	**3.6400E-79**

*The null hypothesis is that there is no relation between these sets of genes and the high and low GC_x_ categories. Significant *p*-values allowing rejection of the null hypothesis (*p* ≤ 0.05) are in bold.*

When considered in totality, up-regulated genes in meiocytes are biased toward low GC_x_ content classes. When we look closely at the smaller number of up-regulated genes with high GC_3_ content, there are energy-based functional gene classes such as electron carrier activity, ATP binding and mitochondria that are enriched and chromosome-specific gene classes such as DNA binding, DNA packaging, and DNA conformation change that are enriched in the up-regulated high GC_3_ genes. These classes were also found to be enriched when analyzing all up-regulated genes in meiocytes, regardless of GC content (Dukowic-Schulze et al., 2014b).

## Discussion

We explored the correlations of genes with instances of a motif found at meiotic DSB hotspots in maize with GC patterns as well as meiotic expression levels. GC-rich motifs with three-nucleotide periodicity underlie hotspots of DSBs that are precursors to recombination between homologous chromosomes. We looked at the genomic landscape of these motifs, including motifs in regions outside of the DSB hotspots that were identified in our dataset. While most DSB hotspots occurred near the transcription start or stop sites, GC_x_ content is, by definition, within the codons. Nevertheless, genes tend to be either high or low GC_x_ as a whole though there is a slight increase in GC content in the 5′–3′ direction (Tatarinova et al., 2010) and GC_x_ determination included bases between the transcription start and stop sites.

These DSB hotspot motifs tend to occur in genes that frequently have high GC_1_, GC_2_, or GC_3_ content. High GC_1_, GC_2_, and GC_3_ content genes are negatively correlated with each other, indicating that the extremely high GC content (≥80%) that occurs in genes with high GC_x_ almost exclusively derive from only one frame.

To incorporate meiotic gene expression, we sequenced RNA from meiocytes (the cells where meiosis takes place) that were just beginning the meiotic process. Specifically, the cells were in the early stages of prophase I, which itself consists of five sequential stages (leptotene, zygotene, pachytene, diplotene, and diakinesis) and is the platform of DSBs and crossovers. Early prophase I includes the formation of DSBs (leptotene), resection and single-end invasion (zygotene), and resolution of some of the DSBs into crossovers (pachytene) and therefore is highly relevant to the generation of novel genetic combinations. Notably, genes that are up-regulated in early meiosis tend to have low GC_x_ and are biased against containing DSB hotspot motifs. Our data thus indicates that genes up-regulated in early meiosis might be protected against DSBs. Our observation that genes that are up-regulated in meiocytes are significantly biased against genes with DSB hotspot motifs makes sense given the environment in early prophase I. Meiotic genes induced during early prophase I, by necessity, need to be genes that are not simultaneously undergoing DSBs, though we emphasize that the presence of a motif does not necessarily indicate the presence of a recombination hotspot. As a key point, our analysis suggests that DSB formation could be targeted to or avoided in specific functional classes, regulated by the periodic GC-rich motif underlying DSBs.

**FIGURE 7:**
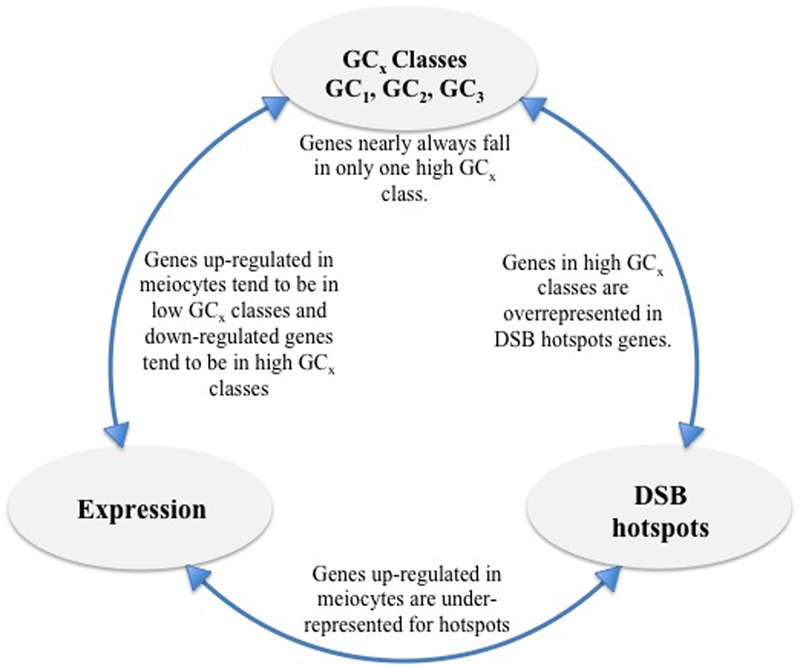
**Overview of findings.** High GC_x_ genes are overrepresented for DSB hotspots and genes down-regulated in meiocytes but underrepresented for genes up-regulated in meiocytes. Individual high GC_x_ classes (GC_1_, GC_2_, and GC_3_) show less overlap than expected given the number of genes in each class. Genes up-regulated in meiocytes are underrepresented for DSB hotspots.

In general, the low GC_x_ gene classes in our dataset were underrepresented for the DSB hotspot motif (**Table 4**; **Figure 7**) and showed bias toward housekeeping functions. In contrast, we found genes with high GC_x_ that are enriched for more specialized functions, including sexual reproduction, though genes did not appear to be specific to meiotic recombination. Possibly, these latter genes are needed during other stages of development or meiosis, and not around the time when DSBs are generated, but benefit from recombination-driven evolution.

GC bimodality refers to the occurrence of two classes of genes, distinguished by their GC content. They have been reported in monocot but not in dicot plants (Clément et al., 2014). To date, there have been several reports explaining the differences in relative distribution of genes based on GC_3_ between monocots and dicots. Additionally, pronounced differences were observed in GC_3_ between close relatives in plant families. For example, a comparative study between *A. thaliana, Raphanus sativus, Brassica rapa*, and *Brassica napus* revealed that the GC_3_ values of *R. sativus, B. rapa*, and *B. napus* are on an average 5% higher than that of *A. thaliana* orthologs (Villagomez and Kuleck, 2009; Tatarinova et al., 2010). Further, many plants, including grasses are prone to genome duplications, which provides redundancy that can relax selection pressure for individual genes though genome-wide some stabilization must occur after a polyploidy event. Nevertheless, the flexibility provided by redundancy that occurs after polyploidization may enable the evolution of genomic regions that have varied recombination rates and GC contents.

Degeneracy in the genetic code in the third codon position gave rise to the wobble hypothesis (Crick, 1966) which has been invoked to explain the bimodal GC_3_ distribution of genes in maize and other monocots (Tatarinova et al., 2010). In the wobble position, tRNA modifications allow pairing of tRNA with multiple codons, allowing multiple codons to code for a single amino acid (reviewed in Agris et al., 2007). This degeneracy of the genetic code gives freedom, mainly in the third codon, for GC shifts to take place.

While the wobble hypothesis is often only applied to the third codon position, there is degeneracy in the genetic code in the first and second positions of the codon as well, though much less than is seen in the third position (**Supplementary Table 1**). Given the much larger amount of wobble in the third codon position compared to the first and second, however, it is surprising that the size of the peak with high GC_1_ content genes is nearly two-thirds that of the corresponding high GC_3_ peak in our dataset of DSB hotspot motif containing genes (**Figure 3**; **Table 1**). Further, the amount of wobble in the first and second positions is quite similar (**Supplementary Table 1**), yet the second position has only 17% the number of genes with high GC_2_ content compared to GC_1_ (**Figure 3**; **Table 1**). If GC bimodality patterns in maize were enabled simply by the freedom that codon wobble gives for GC shifts, than nearly all of the high GC_x_ genes would be in the high GC_3_ category. The fact that nearly half of the high GC_x_ genes fall into the GC_1_ and GC_2_ categories clearly indicates that the wobble hypothesis, on its own, is insufficient for explaining the GC_x_ bimodality across all three frames.

The ability in many parts of a protein to interchange amino acids with amino acids of similar biochemical properties (Henikoff and Henikoff, 1992) might help explain the discrepancy between the numbers of genes in each high GC_x_ class and the codon position’s corresponding wobble. Substitution of amino acids of similar biochemical properties may allow a genome to shift GC content at all the codon positions. More work needs to be done to test this hypothesis and discover its effect on the varying sizes of the high GC_x_ classes across all genes but is not in the scope of this work.

The importance of GC_x_ bimodality is underscored by its maintenance through mutational pressure and selective restraint though the mechanism is yet unknown (Liu et al., 2012). The propensity for recombination to increase GC content (**Figure 1**) may be a contributing mechanism of positive reinforcement for maintaining genes in high GC_x_ content and DSB hotspot pools (Galtier et al., 2001). Further, the typically high evolutionary rates associated with high GC_x_ genes (Tatarinova et al., 2010) could be due to their association with the GC-rich DSB motifs, leading to increased rates of recombination. Genetic recombination is crucial to an organism’s ability to survive, adapt, and evolve. Genetic recombination occurs through crossing over during meiosis and, furthermore, gene conversion occurs with a frequent GC bias in the case of non-crossovers.

DSBs were assayed using ChIP-seq targeting the RAD51 gene (He et al., 2013), which binds to DSBs during zygotene, resulting in the discovery of a maize DSB hotspot motif (He et al., in review). Given the three nucleotide-based GC periodicity found in the DSB hotspot motifs and the documented GC_3_ bias found in maize, it was natural to look for correlations between the two. High correlation was found between genes containing DSB hotspot motifs and GC_3_ levels, both of the motif itself and the containing gene. Interestingly, similar correlations were found for GC_1_ and GC_2_ levels. Indeed, 70% of all genic DSB hotspots fall into high GC_x_ genes, a remarkable and significant proportion given that only one-quarter of genes overall fall into the high GC_x_ category. Even more intriguingly, genes with high GC_2_ motifs make the biggest contribution, although high GC_2_ genes are far less prevalent than GC_1_ or GC_3_ genes (**Figures 5C** and **6B**). Further work needs to be done in order to determine whether there is a direct or secondary relationship between copies of the motif, double-strand breaks, and, by extension, recombination. Nevertheless, this data suggests intriguing hypotheses that imply a role for recombination in maintaining bimodal GC_x_ distributions that cannot fully be explained by the wobble hypothesis.

## Conclusion

Sexual reproduction coupled with meiotic recombination is an important evolutionary strategy for generating genetic diversity crucial to adaptation and evolution of species. As the precursors to crossovers and non-crossovers, DSBs and their locations are critical to understanding recombination processes and biases. Here we showed that DSB hotspot motifs have a strong tendency to occur in genes with high GC_x_ content. These genes have extremely high GC content in one frame, have high evolutionary rates, and are biased toward SOS-functional classes, implicating support for adaptation in these genes. Further, genes up-regulated in meiocytes have a strong bias against high GC_x_ classes and DSB motifs, indicating the importance of these genes and their acquisition of protective strategies against DSBs. Intriguingly, wobble bases and degeneracy in the genetic code are not sufficient for explaining the high GC_x_ classes and their sizes across all three frames. The presence of the three nucleotide-based periodic GC-rich motifs underlying double strand hotspot motifs may provide a first glimpse at additional selection pressure driving the generation of functionally biased high GC_x_ classes in maize and related organisms. More work needs to be done to determine whether the motif provides recognition for the DSB machinery, thus providing a possible mechanism promoting high GC_x_ content.

## Author Contributions

AS, SD-S, and JM analyzed the data and wrote the manuscript. MK, KE, OO, NG, TR, and MG helped with data analysis. MW, YH, QS, and WP performed experiments to identify DSB hotspot motifs and provided data. WP and CC edited the manuscript. WP, CC, SK, JP and JM are investigators on the NSF IOS-1025881 grant of which this manuscript is a part.

## Conflict of Interest Statement

The authors declare that the research was conducted in the absence of any commercial or financial relationships that could be construed as a potential conflict of interest.

## Supplementary Material

SUPPLEMENTAL TABLE 1 **Degeneracy of the genetic code in the first and second codon positions**.
